# Spatial and Temporal Phylogeny of Border Disease Virus in Pyrenean Chamois (*Rupicapra p*. *pyrenaica*)

**DOI:** 10.1371/journal.pone.0168232

**Published:** 2016-12-29

**Authors:** Camilla Luzzago, Erika Ebranati, Oscar Cabezón, Laura Fernández-Sirera, Santiago Lavín, Rosa Rosell, Carla Veo, Luca Rossi, Serena Cavallero, Paolo Lanfranchi, Ignasi Marco, Gianguglielmo Zehender

**Affiliations:** 1 Department of Veterinary Medicine, University of Milan, Milano, Italy; 2 Centro di Ricerca Coordinata Epidemiologia e Sorveglianza Molecolare delle Infezioni—EpiSoMI, University of Milan, Milano, Italy; 3 Department of Biomedical and Clinical Sciences “L.Sacco”, University of Milan, Milano, Italy; 4 Servei d'Ecopatologia de Fauna Salvatge, Departament de Medicina i Cirurgia Animals, Universitat Autònoma de Barcelona, Bellaterra, Barcelona, Spain; 5 Centre de Recerca en Sanitat Animal (CReSA, IRTA-UAB), Campus de la Universitat Autònoma de Barcelona, Bellaterra, Barcelona, Spain; 6 Departament d’Agricultura, Alimentació i Acció Rural, Generalitat de Catalunya, Barcelona, Spain; 7 Department of Veterinary Sciences, University of Torino, Grugliasco, Torino, Italy; 8 Department of Public Health and Infectious Diseases, Section of Parasitology, Sapienza University of Rome, Roma, Italy; Universidade de Aveiro, PORTUGAL

## Abstract

Border disease virus (BDV) affects a wide range of ruminants worldwide, mainly domestic sheep and goat. Since 2001 several outbreaks of disease associated to BDV infection have been described in Pyrenean chamois (*Rupicapra pyrenaica pyrenaica*) in Spain, France and Andorra. In order to reconstruct the most probable places of origin and pathways of dispersion of BDV among Pyrenean chamois, a phylogenetic analysis of 95 BDV 5’untranslated sequences has been performed on chamois and domestic ungulates, including novel sequences and retrieved from public databases, using a Bayesian Markov Chain Monte Carlo method. Discrete and continuous space phylogeography have been applied on chamois sequences dataset, using centroid positions and latitude and longitude coordinates of the animals, respectively.

The estimated mean evolutionary rate of BDV sequences was 2.9×10^−3^ subs/site/year (95% HPD: 1.5–4.6×10^−3^). All the Pyrenean chamois isolates clustered in a unique highly significant clade, that originated from BDV-4a ovine clade. The introduction from sheep (dated back to the early 90s) generated a founder effect on the chamois population and the most probable place of origin of Pyrenean chamois BDV was estimated at coordinates 42.42 N and 1.9 E. The pathways of virus dispersion showed two main routes: the first started on the early 90s of the past century with a westward direction and the second arise in Central Pyrenees. The virus spread westward for more than 125 km and southward for about 50km and the estimated epidemic diffusion rate was about 13.1 km/year (95% HPD 5.2–21.4 km/year). The strong spatial structure, with strains from a single locality segregating together in homogeneous groups, and the significant pathways of viral dispersion among the areas, allowed to reconstruct both events of infection in a single area and of migrations, occurring between neighboring areas.

## Introduction

Pestiviruses are characterized by a high degree of genetic variability and the genus comprises four approved species, namely Border disease virus (BDV), Bovine viral diarrhoea virus type 1 (BVDV-1) and type 2 (BVDV-2), and Classical swine fever virus (CSFV) [1], traditionally classified according to the host species. In the last decades, identification and genetic characterization of pestivirus strains in different animal species revealed an extensive interspecies transmission among both domestic [2,3,4] and wild ungulates [5,6], showing a low host specificity and a wide host range. Besides the interspecies transmission of known genetic variants, several newly emerged pestivirus were detected in livestock [7,8] and in wild ruminant populations [9]. Genetic changes of viruses can lead to alterations of virulence, as recently observed with the Bungowannah pestivirus with high mortality in pigs [10,11] and the emergence of ovine pestiviruses closely related to CSFV in Tunisia and Spain [7,8]. Phylogenetic analysis of pestiviruses is therefore crucial to classify novel viruses and to study evolutionary dynamics, clarifying the relationships between genetic diversity and its temporal-spatial distribution in animal populations.

BDV affects a wide range of ruminants worldwide, mainly domestic sheep and goat, and segregates into at least seven phylogenetic groups [12], namely BDV-1 to BDV-7. Moreover isolates from Tunisia [8], Turkey [13] and Switzerland [14] possibly form further groups.

Concerning pestivirus in chamois, several outbreaks of disease associated to BDV-4 infection have been described in Pyrenean chamois (*Rupicapra pyrenaica pyrenaica*) in Spain, France and Andorra since 2001 [15]. These outbreaks have decimated several Pyrenean chamois populations, with mortalities ranging from 40% to 85% [16]. After the severe BDV-4 outbreaks, different epidemiological scenarios have appeared in the Pyrenees, having a prolonged negative impact on host population dynamics in some areas [17]. Recently, a simulation approach confirmed the ability of BDV-4 to drive chamois populations [18]. In respect to BDV in Alpine chamois (*R*. *rupicapra rupicapra)*, the genetic variant circulating in sheep BDV-6 has been recently detected in a single animal in French Southern Alps, where the chamois population showed seroprevalence of 38.7% with no mortality or clinical diseases [19]. Moreover a genetic variant of livestock origin has been observed in a comprehensive investigation on wild ruminants in Switzerland, with a sporadic frequency of infections detecting a single chamois BVDV-1h positive [20].

Pestivirus seroprevalence in other Alpine countries ranged from negative results in Austria [21] to sporadic, until to a 25.5% value in Italy, without outbreaks mortality [22,23,24].

In order to reconstruct the most probable places of origin and pathways of dispersion of the BDV-4 causing the recent epidemic among Pyrenean chamois, a phylogenetic analysis of BDV 5’untranslated region (UTR) sequences of chamois and domestic ungulates (including novel and retrieved sequences from public databases) has been performed by using a Bayesian framework allowing the spatial–temporal reconstruction of the evolutionary dynamics of highly variable viruses [25].

## Materials and Methods

### Samples and sequence data set

The BDV positive samples and the derived 5’UTR sequences from chamois were selected on the basis of the following criteria: known locality where the strain was isolated and sampling dates. A total of 51 chamois BDV sequences were available, 10 of which were novel Pyrenean chamois sequences and 41 were retrieved from public database, encompassing an alignment of 234 nucleotides (http://www.ncbi.nlm.nih.gov/nuccore/?term=pestivirus+chamois, accessed last time at 29-2-2016). On the whole, 50 sequences were from Pyrenean chamois and one from Alpine chamois, identified in French Alps [19], and the sampling dates ranged from 1996 to 2011.

BDV sequences from domestic ungulates were also retrieved from public database, restricting the geographic localization to Spain and France. A total of 44 BDV 5’UTR sequences were available, encompassing an alignment of 234 nucleotides, 43 of which were from sheep and one from a pig, identified in Spain [26], and the sampling dates ranged from 1984 to 2007 (http://www.ncbi.nlm.nih.gov/nuccore/?term=border+disease+virus+france; http://www.ncbi.nlm.nih.gov/nuccore/?term=border+disease+virus+spain, accessed last time at 29-2-16). Origins and characteristics of data set BDV strains are summarized in Tables 1 and 2 from chamois and domestic ungulates, respectively.

**Table 1:** Accession numbers, references, localities and collection years of the BDV chamois sequences included in the data set.

Id. analysis	Accession no.	Locality	Georeferenced data	Year	Reference
Can1@02	AY738080	Andorra	yes	2002	[27]
Cpa1@02	AY641529	Alt Pallars	yes	2002	[28]
Car1@05	AM765800	Aran	yes	2005	[29]
Car2@05	AM765801	Aran	yes	2005	[29]
Car3@05	AM765802	Aran	yes	2005	[29]
Car4@05	AM765803	Aran	yes	2005	[29]
Car5@06	AM765804	Aran	no	2006	[29]
Car6@06	AM765805	Aran	yes	2006	[29]
Car7@06	AM765806	Aran	yes	2006	[29]
Car8@06	AM765807	Aran	yes	2006	[29]
Cce1@04	AM905930	Cerdanya	yes	2004	[9]
Cce2@05	AM905931	Cerdanya	yes	2005	[9]
Cce3@05	AM905932	Cerdanya	yes	2005	[9]
Cce4@05	AM905933	Cerdanya	yes	2005	[9]
Cca2@05	AM905919	Cadí	no	2005	[9]
Cca3@05	AM905920	Cadí	no	2005	[9]
Cca4@05	AM905921	Cadí	yes	2005	[9]
Cca5@06	AM905922	Cadí	yes	2006	[9]
Cca6@06	AM905923	Cadí	yes	2006	[9]
Cca1@06	AM905918	Cadí	yes	2006	[30]
Cca7@06	AM905924	Cadí	yes	2006	[30]
Cca8@06	AM905925	Cadí	yes	2006	[30]
Cca9@06	AM905926	Cadí	yes	2006	[30]
Cca10@06	AM905927	Cadí	yes	2006	[30]
Cca11@06	AM905928	Cadí	yes	2006	[30]
Cca12@06	AM905929	Cadí	yes	2006	[30]
Cca13@06	FN397676	Cadí	yes	2006	[30]
Cfr1@96	FN691777	Freser-Setcases	yes	1996	[31]
Cfr2@96	FN691778	Freser-Setcases	no	1996	[31]
Car9@08	HE818617	Aran	yes	2008	[17]
Car10@08	HE818618	Aran	yes	2008	[17]
Car11@11	HE818619	Aran	yes	2011	[17]
Car12@11	HE818620	Aran	yes	2011	[17]
Car13@11	HE818621	Aran	yes	2011	[17]
Car14@11	HE818622	Aran	no	2011	[17]
Can2@09	HE615083	Andorra	yes	2009	[32]
Can3@09	HE615084	Andorra	yes	2009	[32]
Can4@09	HE615085	Andorra	yes	2009	[32]
Cpa2@02	LT629121	Alt Pallars	yes	2002	Present study
Cpa3@02	LT629122	Alt Pallars	yes	2002	Present study
Cpa4@02	LT629123	Alt Pallars	yes	2002	Present study
Cpa5@02	LT629124	Alt Pallars	yes	2002	Present study
Cpa6@02	LT629125	Alt Pallars	yes	2002	Present study
Cpa7@02	LT629126	Alt Pallars	yes	2002	Present study
Cor6@04	EU477593	Ariege	no	2004	not available
C4606@06	EU637005	Ariege	no	2006	not available
Cri1@09	LT629127	Alta Ribagorça	yes	2009	Present study
Cri2@09	LT629128	Alta Ribagorça	yes	2009	Present study
Cri3@09	LT629129	Alta Ribagorça	yes	2009	Present study
Cri4@09	LT629130	Alta Ribagorça	no	2009	Present study
CFSA1@11*	KC859383	French Alpes	no	2011	[19]

* sequence from Alpine chamois, all the others are from Pyrenean chamois

**Table 2:** Accession numbers, references, localities and collection years of domestic ungulates BDV sequences included in the data set.

Id. analysis	Accession no.	Locality	Year	Reference
Oal1@99	AY159513	Spain Alava	1999	[7]
Oal2@01	AY159516	Spain Alava	2001	[7]
Oal3@01	AY159517	Spain Alava	2001	[7]
Oal4@01	AY159515	Spain Alava	2001	[7]
Ole2@02	DQ361071	Spain Leon	2002	[33]
Ole3@01	DQ361072	Spain Leon	2001	[33]
Obu4@02	DQ361068	Spain Burgos	2002	[33]
Oza1@02	DQ361070	Spain Zamora	2002	[33]
Ocu1@01	DQ361073	Spain Cuenca	2001	[33]
Obu2@02	DQ361069	Spain Burgos	2002	[33]
Obu3@02	DQ361067	Spain Burgos	2002	[33]
Ote1@04	DQ275625	Spain Teruel	2004	[34]
Ote2@04	DQ275623	Spain Teruel	2004	[34]
Ote3@04	DQ275624	Spain Teruel	2004	[34]
Ole1@01	DQ275626	Spain Leon	2001	[34]
Obu1@02	DQ275622	Spain Burgos	2002	[34]
Opa1@06	EF694003	France PACA	2006	[35]
Opa2@06	EF694002	France PACA	2006	[35]
Opa3@06	EF694001	France PACA	2006	[35]
Opa4@06	EF694000	France PACA	2006	[35]
Opa5@06	EF693999	France PACA	2006	[35]
Omp1@96	EF693998	France Midi-Pyrénées	1996	[35]
Opa6@94	EF693997	France PACA	1994	[35]
Opa7@94	EF693996	France PACA	1994	[35]
Opa8@93	EF693995	France PACA	1993	[35]
Opa9@92	EF693994	France PACA	1992	[35]
Opa10@91	EF693993	France PACA	1991	[35]
Opa11@90	EF693992	France PACA	1990	[35]
Opa12@90	EF693991	France PACA	1990	[35]
Opa13@90	EF693990	France PACA	1990	[35]
Opa14@90	EF693989	France PACA	1990	[35]
Oaq1@89	EF693988	France Aquitaine	1989	[35]
Opa15@89	EF693987	France PACA	1989	[35]
Opa16@85	EF693986	France PACA	1985	[35]
Oce1@85	EF693985	France Centre	1985	[35]
OAV1@84	EF693984	France Midi-Pyrénées	1984	[35]
Opa17@91	EF988632	France PACA	1991	[35]
Opa18@91	EF988633	France PACA	1991	[35]
Oll1@97	FR714860	Spain Lleida-Catalunya	1997	[36]
Oll2@97	HE999869	Spain Lleida-Catalunya	1997	[36]
Oll3@97	HE999870	Spain Lleida-Catalunya	1997	[36]
Oll4@97	HE999871	Spain Lleida-Catalunya	1997	[36]
Oll5@97	HE999872	Spain Lleida-Catalunya	1997	[36]
Pcl1@07*	HF567456	Spain Castilla Leon	2007	[26]

* sequence from pig, all the others are from sheep

### RT-PCR and sequencing

Viral RNA was extracted from chamois spleen tissues using a commercial RNA extraction kit (Nucleospin Viral RNA Isolation, Macherey Nagel, Düren, Germany). Reverse transcription and PCR assays targeting 5’UTR region of pestivirus were performed using 324 and 326 pan-pestivirus primers [37] following previously described protocols [32]. For each sample, amplicons of the expected size were purified and sequenced using forward and reverse primers by cycle sequencing using a Big Dye Terminator version 3.1 kit and an ABI PRISM 3130xl sequencing device (Applied Biosystems, CA, USA).

All sequences from chamois and domestic hosts were aligned with BDV reference strains, retrieved from GenBank and representative of BDV phylogenetic groups according to [12], using CLUSTALW (integrated within the Bio-Edit sequence editor, freely available at http://www.mbio.ncsu.edu/BioEdit/bioedit.html). Phylogeny was preliminary estimated by the neighbor-joining algorithm (NJ) and the maximum likelihood (ML) method.

#### Ethics Statements

This study is part of a project (reference: CGL2006-11518/BOS, CGL2009-09071/BOS, CGL2012-40057-C02-01, FAU 2006-00007-C02-02 and FAU2008-00017-C02-01) from the Spanish Government which aims to study the epidemiology of pestiviruses in wild and domestic animals from Catalonia. The sampling system used in the present study was approved by the Government in the framework of the project cited above. The chamois included in the present study were not sacrificed for our research purposes. The harvested animals have been legally hunted (shot) in their own habitat by authorized gamekeepers and hunters. Samples were obtained from hunted dead animals and diseased animals found dead. No protected species were sampled. The animals were collected in different National Hunting Reserves (NHR) (property of the Government), by rangers employed by the Catalan Government within the framework of an annual hunting plan approved by the Departament d’Agricultura, Ramaderia, Pesca, Alimentació i Medi Natural of the Generalitat de Catalunya (DARPAMN -the Regional authority in charge of livestock and wildlife management-) and no ethics committee approval was necessary. The choice of the animals to hunt was strictly supervised by the rangers. The sampling was performed within the framework of an official agreement between the DARPAMN and Universitat Autònoma de Barcelona. The main aim of the agreement is the surveillance of wildlife diseases from the region of Catalonia, in Spain. After the sampling, samples were delivered to Universitat Autònoma de Barcelona laboratory by government employers.

### Likelihood mapping

The phylogenetic signal of each sequence dataset was investigated by means of the likelihood-mapping analysis of 10,000 random quartets generated using TreePuzzle. All of the three possible unrooted trees for a set of four sequences (quartets) randomly selected from the dataset were reconstructed using the maximum likelihood approach and the selected substitution model. The posterior probabilities of each tree were then plotted on a triangular surface so that the dots representing the fully resolved trees fell at the corners and those representing the unresolved quartets in the centre of the triangle (star-tree) [38]. Using this strategy, which has been described in detail elsewhere [39], the data are considered unreliable for phylogenetic inference when more than 30% of the dots are in the centre of the triangle.

### Phylogenetic reconstruction

The best-fitting nucleotide substitution model was estimated by means of jModeltest [40], and selected a TrN model [41] with gamma-distributed rates among sites.

The phylogenetic tree, model parameters, evolutionary rates and population growth were co-estimated using a Bayesian Markov chain Monte Carlo (MCMC) method implemented in the BEAST v.1.74 package [42].

Statistical support for specific clades was obtained by calculating the posterior probability of each monophyletic clade. As coalescent priors, we compared four simple parametric demographic models (constant population size, and exponential, expansion and logistic population growth) and a piecewise-constant model, the Bayesian skyline plot (BSP) under both a strict and a relaxed (uncorrelated log-normal) clock [42].

Two independent MCMC chains were run for 50 million generations with sampling every 5,000th generation, and were combined using the LogCombiner 1.74 included in the BEAST package. Convergence was assessed on the basis of the effective sampling size (ESS) after a 10% burn-in using Tracer software version 1.5 (http://tree.bio.ed.ac.uk/software/tracer/). Only ESS’s of ≥ 200 were accepted.

Uncertainty in the estimates was indicated by 95% highest posterior density (95% HPD) intervals, and the best-fitting models were selected using a Bayes factor (BF with using marginal likelihoods) implemented in BEAST [43].

In accordance with [44],only values of 2lnBF ≥ 6 were considered significant.

The trees were summarised in a target tree using the Tree Annotator program included in the BEAST package, choosing the tree with the maximum product of posterior probabilities (maximum clade credibility) after a 10% burn-in.

The time of the most recent common ancestor (tMRCA) estimates were expressed as mean and 95% HPD years before the most recent sampling dates, corresponding to 2011 in this study.

### Bayesian phylogeographic analyses

#### Discrete state phylogeography

**Fig 1:**
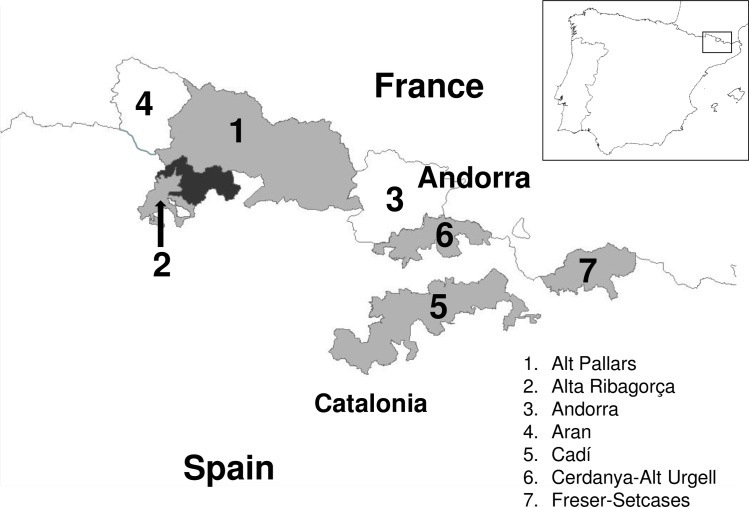
Study area. Map of northeastern Spain showing the distinct geographic areas of Pyrenean chamois BDV sampling location.

The continuous-time Markov Chain (MCC) process over discrete sampling locations implemented in BEAST [25] was used for the geographical analysis, implementing the Bayesian Stochastic Search Variable Selection (BSSVS) model which allows the diffusion rates to be zero with a positive prior probability. Comparison of the posterior and prior probabilities that the individual rates would be zero provided a formal BF for testing the significance of the linkages between locations: rates with a BF of >3 were considered well supported and formed the migration pathway. This analysis was performed for Pyrenean chamois BDV dataset (n = 50) assigned to 7 distinct geographic groups, on the basis of the sampling location, corresponding to: Alt Pallars (APA n = 7), Alta Ribagorça (ARI n = 4), Andorra (AND n = 4), Aran (ARA n = 14), Cadí (CAD n = 13), Cerdanya-Alt Urgell (CER n = 6) Freser-Setcases (FRE n = 2) (Fig 1). In preliminary analysis, Ariege strains (n = 2) significantly clustered in Cerdanya-Alt Urgell area and therefore they have been assigned to this area due to limited number of sequences available. Centroids were used as reference coordinates to build discrete geographic groups. The Maximum Credibility Tree (MCC) final tree was visualised using FigTree version 1.4 (available at http://tree.bio.ed.ac.uk/software). The significant migration rates were analysed and visualised using SPREAD, which is available at http://www.kuleuven.be/aidslab/phylogeography/SPREAD.html.

#### Continuous phylogeography

The Pyrenean chamois BDV diffusion process in continuous space has been investigated using Beast, since localities of origin of most of the chamois carcasses were available (Table 1) and georeferenced by latitude and longitude coordinates.

The two unknown coordinates of the internal nodes and the root of the phylogeny, were estimated under a strict Brownian diffusion model compared to two relaxed random walk (RRW) models, relaxing the diffusion rate constancy assumption [45]. The two RRW models assumed respectively a Gamma distribution and a Cauchy distribution of the diffusion rates over the phylogeny. The Bayes factor comparison between models was made by estimating marginal likelihood by path-sampling (PS) and stepping-stone approaches (SS) [46].

Uncertainty in ancestral location estimation was represented by KML polygons delimitating high probability regions [45].

#### Haplotype network analysis

With the aim to infer intraspecies reticulate evolutionary relationships among samples analysed, a Median Joining Network [47] was constructed using PopArt (Population Analysis– http://popart.otago.ac.nz.) based on sequences available in Table 1.

## Results

### Likelihood mapping and root-to-tip regression analysis

The likelihood mapping of 10,000 random quartets showed that more than 75% were distributed at the corners of the likelihood map and 24.3% in the central area, thus indicating that the dataset contained sufficient phylogenetic information (S1 Fig).

### Evolutionary rate estimation

The BF analysis showed that the relaxed clock fitted the data significantly better than the strict clock (2lnBF = 9.8 for relaxed clock). Under the relaxed clock the BF analysis showed that the exponential growth model was better than the other models (2lnBF>14.6). The estimated mean evolutionary rate of the 5’ UTR BDV sequences analysed was 2.9×10^−3^ subs/site/year (95% HPD: 1.5–4.6×10^−3^).

### Bayesian dated tree analysis

**Fig 2:**
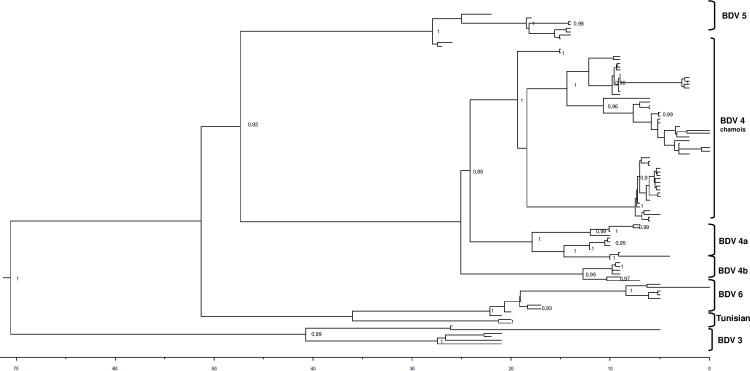
The Maximum clade credibility tree of BDV 5’ UTR sequences from domestic hosts and chamois. The numbers indicate significant posterior probabilities (pp >0.8) of the corresponding nodes and the scale at the bottom of the tree represents the number of years before the last sampling time (2011).

Fig 2 represents the maximum clade credibility tree of BDV sequences from domestic hosts and chamois in France, Andorra and Spain. The ovine sequences clustered in six main clades, supported by posterior probabilities ≥ 0.99, corresponding to the previously described genetic groups, namely BDV-3, BDV-4a, 4b, BDV-5, BDV-6 and Tunisian BDV. The single pig sequence belongs to BDV-4a.

Concerning Pyrenean chamois, all the sequences clustered in a unique clade, supported by posterior probability (pp) of 1, that originated from BDV-4a sheep clade (pp = 0.86). There were also some highly significant subclades (pp = 1) among Pyrenean chamois sequences, clustering three geographical areas in Pyrenees: Eastern area (Freser-Setcases), Central area (Cadí, Cerdanya-Alt Urgell, Ariege) and Western area (Alt Pallars, Alta Ribagorça, Aran and Andorra). The single French Alpine chamois sequence clustered in BDV-6 ovine genetic group as recently reported [19].

### Phylodynamic and phylogeographical analysis of Pyrenean chamois BDV

#### Discrete phylogeography

**Fig 3:**
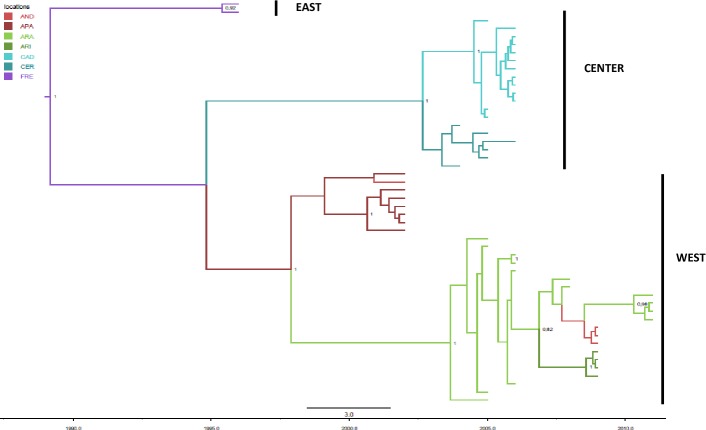
The maximum clade credibility tree of BDV 5’ UTR from Pyrenean chamois. The branches are coloured on the basis of the most probable location of the descendent nodes (Alt Pallars = APA, Alta Ribagorça = ARI, Andorra = AND, Aran = ARA, Cadí = CAD, Cerdanya-Alt Urgell = CER, Freser-Setcases = FRE). The numbers on the internal nodes indicate significant posterior probabilities (pp>0.8), and the scale at the bottom of the tree represents the number of years before the last sampling time (2011). The main geographical clades corresponding to Western, Eastern, and Central Pyrenean areas have been highlighted.

Fig 3 represents the phylogeographic MCC tree of the Pyrenean isolates grouped in discrete geographic category on the basis of the sampling location; the branches of the MCC tree were colored according to the most probable location of their descendent nodes. The analysis of the tree highlighted a significant geographic structure, the strains obtained in a single locality tended to segregate together in homogeneous groups significantly supported by posterior probability values. Strains from Andorra resulted interspersed within Alt Pallars and Aran clades. There were three main clades, corresponding to Western, Eastern, and Central Pyrenean areas previously described (Fig 2) and some significant subclades. The most probable location of the root of the tree was Freser-Setcases, supported by the highest state posterior probability (pp = 0.58 *vs* pp = 0.13 for Alt Pallars, the second most probable location of the root). The tMRCA for the tree root was estimated to be 21.8 years (95% HPD = 15–33.4), corresponding to 1989. Table 3 summarized the tMRCA of the root and internal nodes and the most probable locations for different clades and subclades. In particular the tMRCA shared by the subclades of Western Pyrenees was located in Alt Pallars (pp = 0.63 *vs* 0.23 for Andorra) and the mean tMRCA was 13.1. The MRCA of Central Pyrenees was located in Cerdanya-Alt Urgell (pp = 0.86 *vs* 0.11 for Cadí) and the tMRCA was in 2003.

**Table 3:** Time of the most common ancestor estimates of Pyrenean chamois BDV, credibility interval (95% HPD) of the main clades observed in the MCC tree, with the corresponding most probable locations, and state posterior probability.

CLADE	Subclade	tMRCA^1^	CI tMRCA U^2^	CI tMRCA L^3^	Locality	State pp^4^
ROOT		1989	1978	1996	Freser-Setcases	0,58
EASTERN PYRENEES		1995	1994	1996	Freser-Setcases	0,96
WESTERN PYRENEES		1998	1992	2001	Alt Pallars	0,63
	Alt Pallars	1999	1995	2002	Alt Pallars	0,73
	Aran	2003	2001	2005	Aran	0,92
CENTRAL PYRENEES		2003	2001	2004	Cerdanya-Alt Urgell	0,86
	Cadí	2004	2003	2004	Cadí	0,97

^1^ tMRCa: time of the most common ancestor

^2^ CI tMRCA U: upper credibility interval

^3^ CI tMRCA L: lower credibility interval

^4^ pp: posterior probability

Bayesian phylogeography showed statistically well supported links at the Bayes factor test (BF >3) between the following geographic localities: Alt Pallars and Andorra (BF = 19.57), Aran and Andorra (BF = 64.66), Aran and Alta Ribagorça (BF = 11.94), Cerdanya-Alt Urgell and Cadí (BF = 56.51).

**Fig 4:**
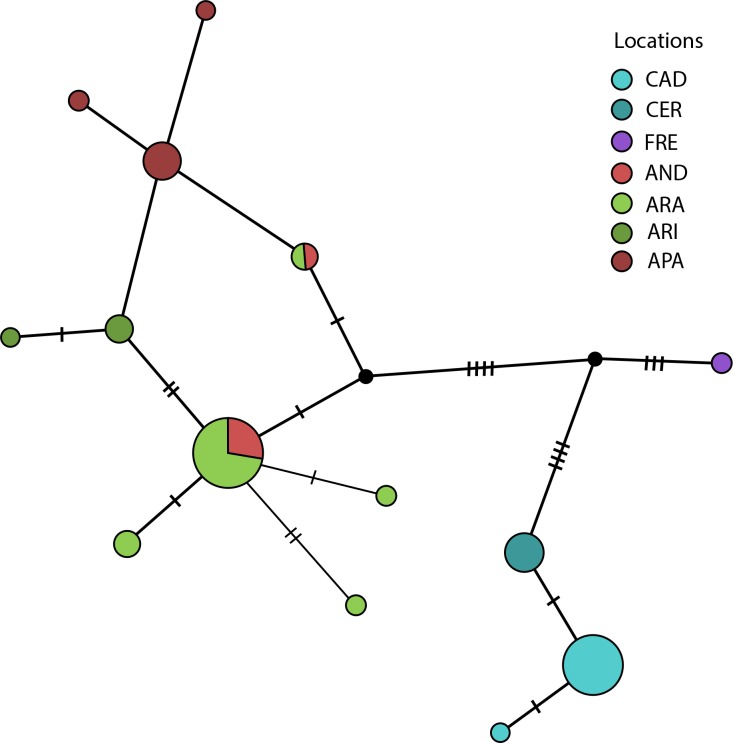
Median-joining network of the 14 haplotypes observed in the BDVs isolates analyzed. Branch lengths are proportional to number of mutations; circles diameter is proportional to haplotypes frequency, transversal bars at branches represent SNPs. The circles are colored on the basis of haplotypes geographical origin (Alt Pallars = APA, Alta Ribagorça = ARI, Andorra = AND, Aran = ARA, Cadí = CAD, Cerdanya-Alt Urgell = CER, Freser-Setcases = FRE).

The Median Joining Network obtained (Fig 4) is congruent with results from Bayesian phylogeny on Pyrenean chamois BDV strains, where strains from a single locality tended to segregate together, with the exception of strains from Andorra: they resulted interspersed within Alt Pallars and Aran in the Bayesian tree clades, while in the reticulate network haplotypes are shared only with Aran. This evidence may explain the paraphyletic position of Andorra strains in Bayesian tree. Moreover, strains from Alt Pallars and Alta Ribagorça could derive from haplotypes of both Andorra and Aran localities.

#### Continuous phylogeography

**Fig 5:**
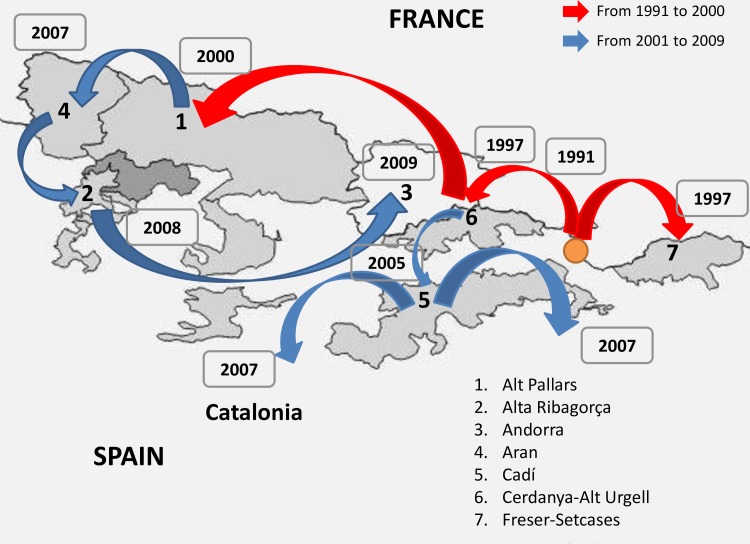
The inferred spatiotemporal dynamics of BDV in Pyrenean chamois. The figure summarize the most significant migration links in the interested Pyrenean area. The putative root of BDV strains is highlighted with a orange circle. More detailed results are reported in supplementary panels (S2 Fig) and at S1 Video.

In order to reconstruct the evolutionary history of the BDV-4 dispersion in a 2 dimensional space a diffusion analysis in continuous space has been performed. A strict Brownian diffusion model, assuming a homogeneous diffusion rates over the phylogeny, was compared with relaxed random walk (RWW) models, assuming different diffusion rates on each branch of the tree. The RWW models gave always better performances than homogenous BD model. In particular a Gamma-distributed RWW diffusion rates model fitted the data better than the other RWW models (Gamma-distribution RWW vs. homogenous BD: 2lnBF = 16.24 by PS and 25.9 by SS; Gamma-distribution vs Cauchy-distribution RWW: 2lnBF = 25.12 and 26.8 respectively). On the basis of the continuous phylogeography, the tree root was placed somewhere between Freser-Setcases and Cerdanya-Alt Urgell (estimated coordinates 42.42 N and 1.9 E) in the early 1990s. Fig 5 summarize the continuous pattern of BDV dispersion in calendar time scale. A more detailed and animated visualization is provided in supplementary panels (S2 Fig) and at S1 Video.

Initially the virus spread to Freser-Setcases and westward, reaching a region between Cerdanya-Alt Urgell and Andorra in 1997. Then it continued its westward diffusion, spreading in a region including Alt Pallars and Aran, between 2000 and 2007, which represented two important radiation points. In particular, from Aran the virus spread southward to Alta Ribagorça and from there it came back eastward, reaching Andorra in 2009. A second principal phylogenetic lineage diffused southward, through Cadí in the early 2005, where the virus was dispersed (radiated) all around in 2005–2007. Globally the virus spread westward for more than 125 km and southward for about 50km. The estimated diffusion rate of the epidemic was about 13.1 km/year (95% HPD 5.2–21.4 km/year).

## Discussion

Identification and genetic characterization of BDV strains from Pyrenean chamois have been performed since the first outbreaks [27], indeed the intensive monitoring of found dead or hunted chamois allowed to collect a large number of strains from different areas in the Pyrenees and therefore to apply advanced phylogenetic analysis. Previous investigations performed phylogenetic analysis using the neighbor-joining (NJ) method and classified Pyrenean chamois strains within BDV-4 genotype [27,28].

In order to reconstruct origin, time of introduction and pathways of dispersion of the Pyrenean chamois BDV genetic variants, a comprehensive collection of publicly available ovine and chamois BDV sequences of Spanish, Andorran and French origin has been analyzed by using a Bayesian framework allowing the spatial–temporal reconstruction of the evolutionary dynamics of highly variable viruses [25].

The evolutionary rate estimated for BDV sequences showed values, between 1.5 and 4.6 substitutions for 1000 nucleotides, in agreement with the range observed for other RNA viruses [48]. Interestingly a similar evolutionary rate was already estimated for BVDV-1 in cattle [49], using the same genomic region, namely 5’-UTR, commonly considered conserved [50], highlighting the evolution of pestivirus in short period of time.

All the Pyrenean chamois isolates cluster in a unique highly significant clade within the BDV-4 genotypes, including chamois sequences from Spain, France and Andorra, in agreement with previous analysis using NJ methods performed on a more restricted dataset [9]. On the whole, BDV-4 includes also Spanish ovine isolates and segregate into three highly significant subclades: BDV-4a, BDV-4b [34] and a third one of the Pyrenean chamois isolates. The phylodynamic and phylogeographical analysis suggests that BDV-4a ovine and Pyrenean chamois clades shared a common ancestor, supporting the hypothesis [51] that the chamois viral genetic variant originated from sheep. In this respect, BDV-4a represents a wide dispersed genetic group in ovine population in different areas in Spain [34].

The entire diversity of Pyrenean chamois BDV genetic group is likely descended from a single introduction from sheep that has generated a founder effect on the chamois population due to intraspecies spread and spatial dispersion.

Phylogenetic and network analysis showed that the Pyrenean chamois BDV isolates analyzed segregated on a geographical basis into three highly significant clades corresponding to the East, Central and West Pyrenees. Given this strong spatial structure of the isolates and in order to have a complete reconstruction of the origin and pathway of dispersion of the BDV epidemic, we initially employed a discrete phylogeography approach. In this reconstruction, BDV chamois genetic variant most probably originated between Freser-Setcases and Cerdanya-Alt Urgell, dating back to a time span between 1978 and 1996, with a mean estimation falling in 1989. This period of time confirms the relatively recent emergence of BDV in Pyrenean chamois and is highly consistent with retrospective sero-epidemiological and molecular investigations that reported BDV at least since 1990 and 1995 respectively in Spanish Pyrenees [31] and in French Pyrenees [52].

Afterwards, two highly divergent Pyrenean clades originated: the Western Pyrenean dating back to late 1990s, originating from Alt Pallars and reaching the westernmost part of the spreading area (Aran) and Alta Ribagorça in 2011, and the Central-Southern Pyrenean originating in early 2000s from Cerdanya-Alt Urgell and reaching Cadí only later.

The main difficulty in the discrete analysis was to discriminate the different localities in such a restricted area. In order to solve this problem, the centroid positions of the recovered carcasses have been used, but even in this case the pathway of the diffusion of the infection was only inadequately described by the significant rates between discrete localities. This might depend on the movements of wild animals in mountain territory in relation to ecological, ethological factors and also human management. Moreover, it is well known that discrete phylogeography is strongly influenced from the set of observed locations of the sampled isolates. For all these reasons, given that latitude and longitude coordinates were available for each isolate, the phylogeography was more deeply analyzed under a continuous space diffusion model that allowed a more detailed description of the pattern of dispersion of the virus in the Pyrenean area.

The main pathways of dispersion reconstructed by discrete phylogeography have been strongly supported also by the two-dimensional dispersion of BDV in continuous space that showed two main streams, corresponding to the two evolutionary lineages in the tree: the first mainstream had a westward direction since the early 90s and a second stream arise in Cerdanya-Alt Urgell.

The first one spread in Alt Pallars and by about 2007 reached its westernmost area (Aran), then it diverged southward and turned back eastward. In this new pathway of dispersion the infection reached areas previously free of the virus, such as Alta Ribagorça in the South, or areas already affected, such as Alt Pallars. In Andorra, virus dispersion, reported since the early 2000s [27], has been spatial and temporal related to Alt Pallars and Aran areas as suggested by [32]. The pathway of dispersion of isolates among these areas suggests a complex exchange between neighboring areas, supporting the epidemiological evidence of a frequent circulation of BDV in Western Pyrenees [17]. The second stream that arise in Cerdanya-Alt Urgell heads south, reaching Cadí, and dispersing all around.

The estimated diffusion rate of the BDV epidemic (13.1 km/year), similar to rabies virus in established enzootic situations in wildlife animals [53], concur to the observed phylogeographic pattern with a spatial segregation of lineages.

In conclusion, the phylogeographic analysis of Pyrenean chamois BDV strongly suggests a recent introduction from sheep (dated back to the early 90s) that generated a founder effect within the chamois population. Moreover the strong spatial structure, with the strains obtained in a single locality that tended to segregate together in homogeneous groups, and the significant pathways of viral dispersion among the areas allowed to reconstruct both events of infection in a single area and migrations events, occurring between neighboring areas. Finally, this study highlights the importance of using continuous space phylogeography, when geographical coordinates of each isolate are available, in order to obtain a detailed reconstruction of the virus dispersion pattern.

## Supporting Information

S1 Fig Likelihood map of the BDV 5’UTR sequences. Each dot represents the likelihoods of the three possible unrooted trees per quartet randomly selected from the data set. The numbers indicate the percentage of dots in the centre of the triangle. Fully resolved trees fell at the corners and the unresolved fell at the centre area.
(TIF)

S2 Fig The inferred spatiotemporal dynamics of BDV in Pyrenean chamois. The panels provide the continuous pattern of BDV dispersion for 1991 (A), 1997 (B), 2000 (C), 2007 (D) and 2011 (E). Lines represent MCC phylogeny branches projected on the map, based on satellite pictures made available in Google Earth (http://earth.google.com). Uncertainty in ancestral location estimation was represented by KML full coloured polygons (green gradient) delimitating high probability regions. Bright green and blue circles (D) highlight the two principal BDV lineages dispersion areas.
(PPTX)

S1 Video Animated visualization of the continuous pattern of Pyrenean chamois BDV dispersion. (MP4)
