# Supplementary figures and images for: Spatial and Temporal Phylogeny of Border Disease Virus in Pyrenean Chamois (Rupicapra p. pyrenaica)
Source: PLoS One. 2016 Dec 29;11(12):e0168232. doi: 10.1371/journal.pone.0168232 (PMC5199066; doi:10.1371/journal.pone.0168232)

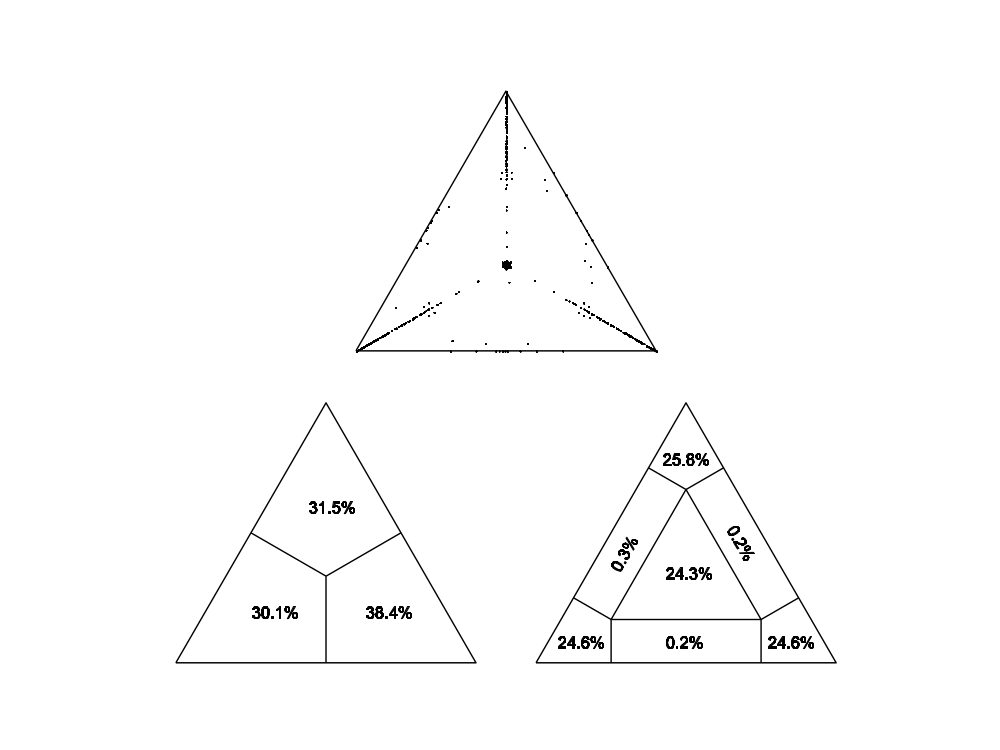

Supplement: S1 Fig — Each dot represents the likelihoods of the three possible unrooted trees per quartet randomly selected from the data set. The numbers indicate the percentage of dots in the centre of the triangle. Fully resolved trees fell at the corners and the unresolved fell at the centre area. (TIF) [file pone.0168232.s001.tif]

## Slide 1
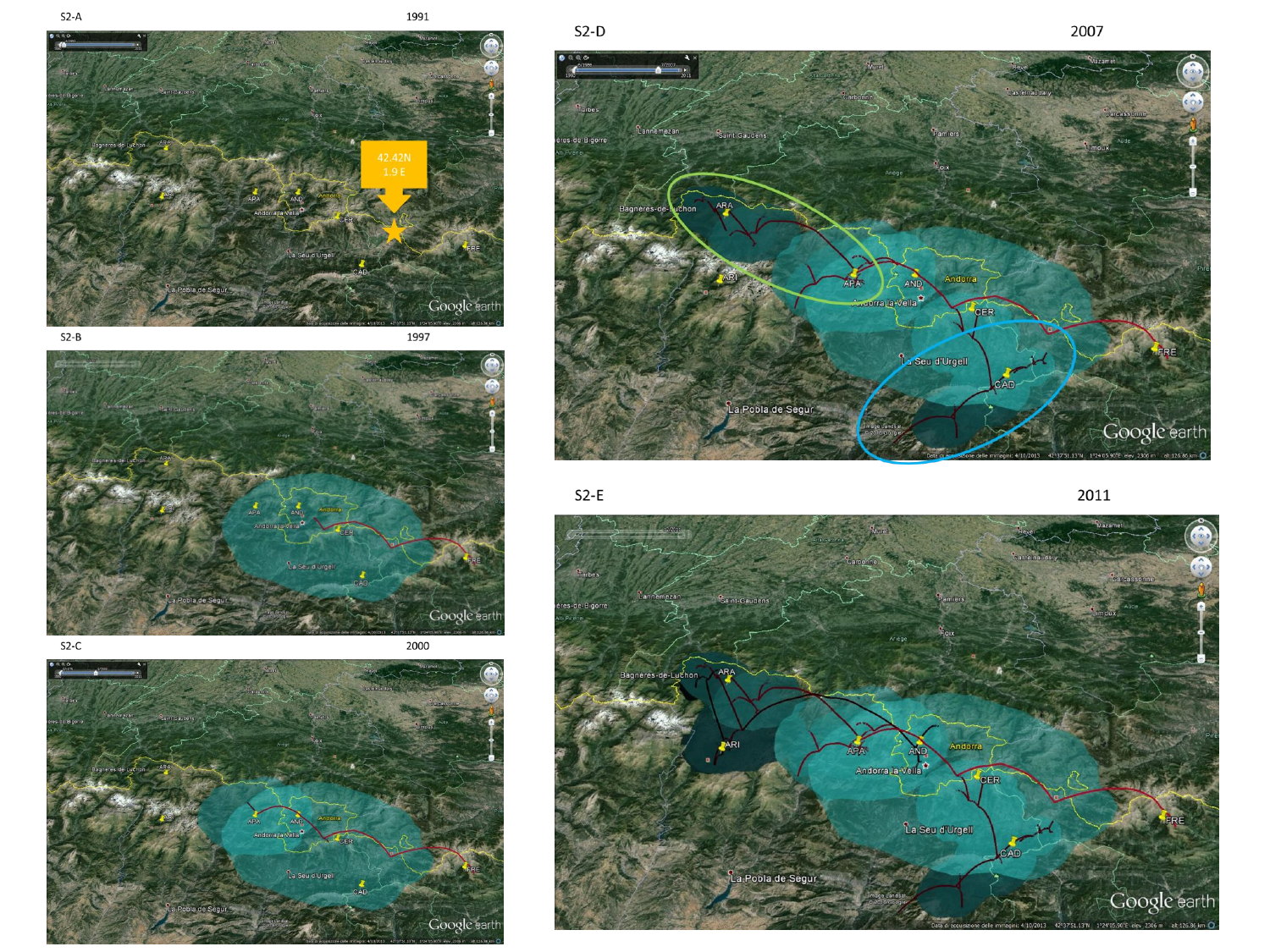

Supplement: S2 Fig — The panels provide the continuous pattern of BDV dispersion for 1991 (A), 1997 (B), 2000 (C), 2007 (D) and 2011 (E). Lines represent MCC phylogeny branches projected on the map, based on satellite pictures made available in Google Earth (http://earth.google.com). Uncertainty in ancestral location estimation was represented by KML full coloured polygons (green gradient) delimitating high probability regions. Bright green and blue circles (D) highlight the two principal BDV lineages dispersion areas. (PPTX) [file pone.0168232.s002.pptx]
